# IS VARIABILITY OF FREE-LIVING ACTIVITY ASSOCIATED WITH PHYSICAL AND MENTAL FATIGABILITY IN OLDER ADULTS?

**DOI:** 10.1093/geroni/igz038.867

**Published:** 2019-11-08

**Authors:** Jessica L Graves, Robert T Krafty, Jaroslaw Harezlak, Eric J Shiroma, Nancy W Glynn

**Affiliations:** 1 University of Pittsburgh, Department of Epidemiology, Pittsburgh, Pennsylvania, United States; 2 Department of Biostatistics, Graduate School of Public Health, University of Pittsburgh, Pittsburgh, Pennsylvania, United States; 3 Department of Epidemiology and Biostatistics, School of Public Health, Indiana University, Bloomington, Indiana, United States; 4 Laboratory of Epidemiology and Population Science, National Institute on Aging, Bethesda, Maryland, United States

## Abstract

Greater fatigability in older adults may be moderated by physical activity (PA). However, what features of PA timing are most strongly related to fatigability remains unknown. We examined the relationship between variability of free-living activity patterns and perceived physical and mental fatigability using the Pittsburgh Fatigability Scale (PFS, 0-50pts, higher=greater fatigability) in older adults from the Developmental Epidemiologic Cohort Study (DECOS, n=57, age=70-91yrs, 61% female). We assessed PA using ActiGraph GT3X+ over 7 days. Mean activity, standard deviation (SD) of mean activity across days, and relative activity [(mean at each bin)/(total mean)] were calculated across 24-hours in 4-hour bins , adjusting for estimated rise-time. Lower SD of PA from 0-4 hours after rising was associated with greater PFS physical scores (r=-0.27, p=0.05). No measures of PA correlated with PFS mental scores. In older adults with lower physical fatigability, associations with greater variability in activity may indicate larger energy reserves.

